# Factors associated with quality of life in elderly undertaking literacy programs

**DOI:** 10.1590/S1980-57642014DN82000013

**Published:** 2014

**Authors:** Bruna Rodrigues dos Santos, Sofia Cristina Iost Pavarini, Allan Gustavo Brigola, Fabiana de Souza Orlandi, Keika Inouye

**Affiliations:** 1 Graduanda em Gerontologia/ Departamento de Gerontologia, Universidade Federal de São Carlos, São Carlos SP, Brazil.; 2 Docente/Departamento de Gerontologia, Universidade Federal de São Carlos, São Carlos SP, Brazil.; 3 Mestrando em Enfermagem/ Departamento de Enfermagem, Universidade Federal de São Carlos, São Carlos SP, Brazil.

**Keywords:** cognition, depressive symptoms, quality of life, education

## Abstract

Increased life expectancy has led to a significant number of elderly enrolling on Youth and Adult Education programs (YAE). These individuals leave inactivity and negative aspects of aging in search of opportunities for social inclusion.

**Objective:**

To evaluate the influence of sociodemographic factors and depressive and cognitive symptoms on quality of life (QL) of elderly attending the YAE of São Carlos city in São Paulo state.

**Methods:**

A descriptive and quantitative study approved by the Research Ethics Committee of São Carlos Federal University was conducted. The sample comprised all elderly undertaking the YAE literacy program in 2012. The instruments used were the Mini-Mental State Examination (MMSE), Geriatric Depression Scale (GDS), WHOQOL-bref and WHOQOL-old, and a sociodemographic instrument.

**Results:**

We interviewed 23 elderly, predominantly females (91.3%) in the early stages of old age (69.6%). The number of years of YAE study showed no correlation with cognition scores obtained on the MMSE or with QL domains. However, scores on the GDS had a moderate inverse relationship with total scores for the Physical (p<0.01), Sensory Functioning (p<0.05), Independence (p<0.01), Past, Present and Future Activities (p<0.05), Social Participation (p<0.01), and Intimacy (p<0.05) QV domains, and a strong inversely proportional relationship with the Social Relationships QV domain (p<0.01). Scores attained on the MMSE showed a moderate and direct relationship with total scores on the Independence QL domain (p=0.001).

**Conclusion:**

Elderly on literacy programs have average quality of life scores. Several QL domains are influenced by depression and cognitive symptoms.

## Abstract (pt)

O aumento da expectativa de vida trouxe um significativo contingente de idosos aos programas de Educação de Jovens e Adultos (EJA). Essas pessoas deixam a inatividade e os aspectos negativos do envelhecimento e buscam novas oportunidades de inclusão social.

**Objetivo:**

Avaliar a relação de fatores sociodemográficos e sintomas depressivos e cognitivos na qualidade de vida (QV) de idosos que frequentam a EJA do município de São Carlos-SP.

**Métodos:**

Pesquisa descritiva e quantitativa aprovada pelo Comitê de Ética em Pesquisa da Universidade Federal de São Carlos. A amostra foi composta por todos os idosos que estavam no processo de alfabetização na EJA, em 2012. Os instrumentos utilizados foram o Mini-exame do Estado Mental (MEEM), a Escala de Depressão Geriátrica (GDS), o WHOQOL-bref e WHOQOL-old, e um instrumento sociodemográfico.

**Resultados:**

Foram entrevistados 23 idosos, em sua maioria do sexo feminino (91,3%), nos anos iniciais da velhice (69,6%). O número de anos de estudo EJA não apresentou correlação com os escores de cognição obtidos por meio do MEEM e com os domínios de QV. No entanto, a pontuação na GDS teve relação inversa e moderada com os escores totais dos domínios de QV Físico (p<0,01), Funcionamento do Sensório (p<0,05), Autonomia (p<0,01), Atividades Passadas, Presentes e Futuras (p<0,05), Participação social (p<0,01) e Intimidade (p<0,05) e relação forte e inversamente proporcional com o domínio Relações Sociais (p<0,01). E as pontuações obtidas a partir do MEEM mostrou relação moderada e direta com os escores totais do domínio QV Autonomia (p=0,001).

**Conclusão:**

Idosos em programas de alfabetização têm uma pontuação média de qualidade de vida e alguns domínios da QV são influenciados por sintomas depressivos e cognitivos.

## INTRODUCTION

Literacy programs for adults help to combat prejudice against illiteracy and the elderly. These programs now have a significant number of participants aged 60 years or over. According to the Census of the Brazilian Institute of Geography and Statistics (IBGE), in 2011 in São Paulo State, there were a total of 14,213 participants on classes of the Youth and Adult Education (YAE) system aged 60 years or older, with approximately 70.7% of this number taking classes from 1^st^ to 4^th^ grade.^1^

With regard to age, according to IBGE figures for 2010, 42.6% of Brazilian illiterates were elderly people (approximately six million individuals), almost ten-fold the estimated 650,000 illiterate Brazilians in the 15-24 year-old age band.^2^

Barnes & Yaffe (2011), in a systematic review of potentially modifiable risk factors for Alzheimer's disease (AD), indicated that low educational level is the most prevalent risk factor for AD. Worldwide, about 19% (6.5 million) of AD cases are potentially attributable to low education level. The authors emphasize that a 10% reduction in the prevalence of low education level can potentially reduce the prevalence of AD by more than 500,000 cases worldwide while a 25% reduction could reduce the number of cases by 1.4 million globally.^3^

Education is one of the main ways of overcoming the challenges faced by elderly people in society today, because the learning of new knowledge and opportunities enables them to attain physical and emotional well-being, while the inability to read and write remains a barrier for many.^4,5^

This undeniable reality of conduits and social transformations highlights the importance of ensuring the elderly, besides adequate conditions of health, enjoy good quality of life (QL).

Thus, there is a connection between education and elderly QL, a contemporary theme that is becoming increasingly important in many aspects. According to Rocha and Bartholo (2010), "quality of life encompasses many meanings that reflect knowledge, experiences and values, both individual and collective, and these meanings express the generation, social class and culture which they belong to".^6^

The QL concept is comprehensive in terms of its multidimensional and subjective nature. Although there is no single definition for this, the most used is the concept defined by the Quality of Life Group of the WHO, which defines QL as "the perception that the individual has of their position in life in the culture, context and value system in which they live and in relation to their goals, expectations, standards and concerns".^7^ According to the WHO, as an individual gets older their QL is largely determined by their ability to maintain autonomy and independence.^8^

Studies on QL and aging are a growing subject of research. Although many studies involve health-related QL in the elderly, it is important to emphasize that the concept is multidimensional and should also be related to education.

Cruz (2003), aiming to identify factors responsible for subjective well-being attained by elderly participants from different social groups, noted that individuals with higher satisfaction were those who had higher levels of education.^9^

A study on the quality of life of the elderly (n=257) from the University of the Third Age (U3A) in Poland, using the WHOQOL-bref for quality of life and the Geriatric Depression Scale (GDS) for assessment of depressive symptoms, found that most participants reported they were in good health and had no signs of depressive symptoms. There was influence of the educational level of students on environmental QL, but no effect related to the variables age, sex or marital status. The relationship between subjectively reported diseases and/or discomfort with QL was evident in the physical domain.^10^

Given this century is considered the information age, it is necessary to understand and evaluate the link of QL levels in the population of this age group as connected with the permanent need for elderly education, social action ensuring elderly have access to knowledge, and citizenship.^6^ It is currently held that education forms the basis for the full exercising of human rights and for breaking the cycle of social exclusion, making the teaching mode of YAE important for the elderly in this process.

The aim of this study was to evaluate the relationship between quality of life and depressive symptoms, cognition and time that elderly have been attending YAE education programs in the city of São Carlos - São Paulo state.

## METHODS

A descriptive, correlational study with a cross-sectional design was performed based on the quantitative research method assumptions.

All ethical guidelines were followed. The procedures for data collection were performed after approval of the Research Ethics Committee of São Carlos Federal University - UFSCAR (No. 72055/2012). Data collection was performed after the participants had signed a Consent Form.

**Subjects.** The study was conducted with 23 students aged over 60 years that were in the process of literacy (temping I - 1^st^ to 4^th^ grade) in YAE and who agreed to participate in the study. Of the total participants enrolled, 29 were over 60 years old, 23 of whom agreed to participate in the study. Six seniors were excluded, four because of health problems and two for absence after three attempts at data collection. The students were from different public schools and regions of the city.

**Date collection and analysis procedure.** Data collection was performed from June to August of 2012. Individual interviews previously scheduled with elderly, at the schools where they attended in places reserved for this purpose, were conducted. Data were organized in electronic databases, keyed into a spreadsheet of the Statistical Program SPSS (Statistical Package for Social Science) version 19.0. Subsequently, descriptive statistical analysis was carried out with data presented in table format containing frequency and measures (mean, median, minimum and maximum) and dispersion (standard deviation). Correlational statistics were also performed. The Shapiro-Wilk test was used to verify the data normality. Confidence and significance levels adopted were 95% and 5% (p≤0.05), respectively.

### Instruments for data collection

***Sociodemographics* -** The instrument used was constructed based on the Gerontological Evaluation Protocol of the undergraduate degree course in Gerontology at the Federal University of São Carlos,^11^ collecting information on age, sex, race, income, marital status and time enrolled on the program to characterize the elderly participants.

*Quality of life assessment* - The WHOQOL-bref and WHOQOL-old instruments were used, both validated by the WHO to assess QL in several domains, i.e. individual perceptions in relation to the life context in which they live.

The WHOQOL-bref is a generic tool for assessing overall QL, developed in 1998 to meet the need for a tool for rapid implementation. This is the abbreviated version of the WHOQOL-100, which consists of 26 questions divided into four domains: physical, psychological, social and environmental relationships. From this set, two questions are about general QL and the other 24 represent each of the 24 facets that make up the original instrument. In Brazil, the instrument was validated by Fleck et al. in 2000. The results are obtained by converting the responses into scores for the areas on a scale ranging from 0 (worst) to 100 (best), with no single value that summarizes the entire assessment, resulting in a better or worse general state of QL.^12^

The WHOQOL-old emerged from the WHOQOL group seeking to develop an instrument for assessing QL in elderly, justified by the fact that you cannot simply take the same variables from WHOQOL-100 and WHOQOL-bref and apply them to the elderly population since there are particularities to be evaluated in this age group. Considering the subjective nature of QL and its multidimensional nature, the WHOQOL-old is a modular instrument for assessing QL, validated for use in the Brazilian context that should be applied together with the WHOQOL-100 or WHOQOL-bref. It consists of 24 items from the Likert scale assigned to six facets: sensory functioning, independence, past, present and future activities, social participation, death and dying, and intimacy.^13^

*Assessment of depressive symptoms -* The Geriatric Depression Scale (GDS), 15-item version, created by Sheikh-Yesavage in 1986,^14^ was used. The GDS is a reduced version of the original questionnaire developed by Yesavage et al. in 1983 (30-item version).^15^ The shorter version chosen for this study was previously validated for use in Brazil by Almeida and Almeida (1999). For responses, the respondent must check or state yes or no after each question.^16^ A result of between six and 10 points is indicative of the presence of mild depressive symptoms, while a score greater than or equal to 11 indicates severe depressive symptoms.

*Tracking cognitive changes -* We adopted the Mini-Mental State Examination (MMSE), an instrument used internationally, which provides information about different cognitive dimensions. The MMSE was developed by Folstein et al. in 1975^17^ and translated, adapted and validated for use in Brazil by Bertolucci et al. in 1994.18 It involves different categories of verbal and nonverbal responses which quantify the following cognitive aspects: temporal and spatial orientation, immediate recall, procedural memory, attention, language, and visual-constructive ability. The test consists of 12 items with a maximum score of 30 points. The cut-off score, which means the score that indicates the probable presence of cognitive impairment varies according to the individual's level of education, and averages suggested by Brucki et al. (2003) were used.^19^

## RESULTS

**Elderly characterization.** The elderly were aged from 60 to 79 years old, having a mean age of approximately 67.5 years (±5.92), with a predominance of elderly between 60 and 64 years old.

Of these, 91.3% (n=21) were females. Regarding marital status, the majority were widowed 47.83% (n=11), followed by married 39.13% (n=9).

The majority of the elderly professed the Catholic religion, 69.57% (n=16), regarded themselves of white color, 82.6% (n=19) and had an individual income of 1 to 2 minimum wages, 52.17% (n=12) (Table 1).

**Table 1:** Distribution of elderly students attending youth and adult education programs according to sociodemographic variables. São Carlos, 2012.

Characteristics		n	%
Gender	Female	21	91.3
	Male	2	8.7
Age	60 to 64 years	9	39.13
	65 to 69 years	7	30.44
	70 to 74 years	4	17.39
	75 to 79 years	3	13.04
Marital status	Single	0	0
	Married	9	39.13
	Widowers	11	47.83
	Divorced or separated	3	13.04
Ethnicity	White	19	82.6
	Brown	1	4.35
	Black	3	13.05
Religion	Catholic	16	69.57
	Evangelical	7	30.43
Individual income	No individual income	1	4.35
	Up to 1 minimum wage	7	30.43
	From 1 to 2 minimum wages	12	52.17
	From 2 to 3 minimum wages	2	8.7
	From 3 to 5 minimum wages	1	4.35

Source: Research data.

Regarding family composition, 21.73% (n=5) of the elderly lived only with their spouse; 26.09% (n=6) only with children, 8.7% (n=2) with spouse, children and grandchildren, 8.7% (n=2) with spouse and children, 4.35% (n=1) with children and grandchildren, and 8.7% (n=2) with other family members such as siblings, in-laws or nephews. None of the elderly reported house-hold members without blood ties. However, 21.73% (n=5) of the elderly reported living alone.

Regarding the GDS, 52.18% (n=12) of subjects had signs of depressive symptoms, with 39.13% (n=9) reporting mild depressive symptoms and 13.05% (n=3) severe depressive symptoms.

On the cognitive evaluation, as measured by the Mini-Mental State Examination (MMSE), 13.05% (n=3), participants scored lower than the expected cut-off for schooling level, while 86.95% (n=20) had higher than expected scores.

For the quality of life evaluation using the WHOQOL-bref and WHOQOL-old, the best analyzed domain was Sensory Functioning (M=76.3, ±21.7); and the worst was the Environment (M=63.4, ±15.7) (Table 2), where no single value summarized the entire assessment, resulting in only a definition of a general state of better or worse quality of life.

**Table 2:** Distribution of results by domain area, expressed as lower value, higher value and arithmetic average, according to the WHOQOL-bref and WHOQOLold. São Carlos, 2012.

Variables		Description (n=23)		
		Lower value (minimum)	Higher Value (maximum)	Arithmetic average
WHOQOL-bref	Physical domain	32.15	96.42	66.76 (±15.55)
	Social relationships domain	20.83	95.83	62.85 (±17.4)
	Psychological domain	8.33	100	65.57 (±21.8)
	Environment domain	31.25	87.5	62.36 (±15.66)
WHOQOL-old	Sensory functioning domain	12.5	100	76.35 (±21.73)
	Autonomy domain	12.5	100	67.66 (±21.04)
	Past, present and future activities domain	18.75	93.75	70.65 (±19.44)
	Social participation domain	18.75	93.75	64.67 (±19.73)
	Death and dying domain	0	100	63.04 (±26.1)
	Intimacy domain	0	100	63.31 (±30.87)

Source: Research data.

**Variables associated with quality of life of elderly undertaking literacy programs.** On the correlational analysis between age, time enrolled on the program, depressive symptoms, cognitive symptoms and quality of life (Spearman Correlational Test), the variables age and years of education were not associated with any quality of life domain. However, the score on the GDS had a moderate inverse relationship with total scores on the domains QL Physical (rho= -0.653, p<0.01), Sensory Motor Functioning (rho= -0.472, p<0.05), Independence (rho= -0.599, p<0.01), Past, Present and Future Activities (rho= -0.454, p <0.05), Social Participation (rho= -0.567, p<0.01), and Intimacy (rho= -0.445, p<0.05), and a strong inversely proportional relationship with the Social Relationships domain (rho= -0.896, p<0.01).

The scores attained on the MMSE exhibited a moderate and direct relationship with the total scores on the QL Autonomy domain (r=0.654, p=0.001).

## DISCUSSION

The results showed a female predominance, in the early stages of old age, i.e. from 60 to 64 years old (M=67.5, ±5.92 years old), a phenomenon also shown in the literature.^10,20-22^ Furthermore, the high percentage of elderly living alone is notable. According to the 2010 Census, there are nearly three million seniors living alone in Brazil, representing about 14% of all Brazilians aged 60 or over.^23^ In relation to the years of study of the elderly in Youth and Adult Education, time on the programs ranged from six months to 17 years. This discrepancy occurs because until students have learned how to read and/or write, they remain at grades I and II of YAE. However, no literature data on the time spent on literacy programs for the elderly were found to serve as a comparison with our findings.

For the mood symptoms assessed by the GDS, 52.18% presented depressive symptoms signs, defined as six points or above on the scoring scale. A study that examined the prevalence of depressive symptoms among elderly participants in an Open University for the Third Age showed that, of the 184 people interviewed, only 17 (9.23%) participants scored more than six points on the GDS.^24^

The MMSE scores were correlated with the QL autonomy domain. Kissaki et al. (2012) investigated elderly University of the Third Age (U3A) students and found a significant positive association between education variables and cognitive test performance.^25^

The results also showed that elderly on literacy programs had an average score of between 63.4 and 76.3 on the different QL domains. A study with elderly from an Open U3A found an average score of between 66.5 and 69.4 and a relationship between QL and higher education^10^.

Years of education were not significantly related to any QL domain, as was expected.

An inverse relationship between depressive symptoms and all domains of quality of life was found, except for the psychological, environmental, death and dying domains, in line with findings in the literature.

A study of Farenza et al. (2007) evaluating the quality of life in a group of elderly from Veranopolis, Rio Grande do Sul state, also found results that were inversely proportional, and statistically significant with p<0.01, on all WHOQOL-bref domains when correlated with the GDS.

A Polish study on the quality of life of 257 elderly at a Third Age University, also using the WHOQOL-bref to evaluate quality of life and the GDS to assess depressive symptoms, observed that the occurrence of depressive symptoms negatively affected elderly quality of life on all domains studied, except for the physical domain.^10^

**Final considerations.** Although no significant relationship between quality of life and time of elderly participation in adult education were found (as expected), we can conclude that the elderly on literacy programs have an average score of quality of life and that some quality domains are influenced by depression and cognitive symptoms.

Our results cannot be widely generalized because they only represent the elderly from a small region of Brazil who are on literacy programs. This study has some limitations such as the small number of subjects, although the percentage of elderly respondents was high compared to the total of elderly enrolled on YAE in the town (79.3%). No specific studies on elderly illiterates were found in formal education, which limits comparison of results and discussion. In summary, given the scarcity of literature, further studies on the subject are warranted, such as longitudinal studies to effectively check the relationship between length of study with quality of life. Also, it would be useful to include a control group to better identify the influence of programs on elderly quality of life.

Considering the illiteracy of the elderly population and the consequences for their quality of life, further research in this area is needed.
